# Constant or fluctuating hyperglycemias increases cytomembrane stiffness of human umbilical vein endothelial cells in culture: roles of cytoskeletal rearrangement and nitric oxide synthesis

**DOI:** 10.1186/1471-2121-14-22

**Published:** 2013-04-22

**Authors:** Xianxian Chen, Lie Feng, Hua Jin

**Affiliations:** 1 The First Affiliated Hospital of Jinan University, Guangzhou, Guangdong, China; 2 Department of Chemistry, Jinan University, Guangzhou, Guangdong, China

## Abstract

**Background:**

Previous studies have implicated continuous or intermittent hyperglycemia in altered endothelium-derived nitric oxide (NO) synthesis. NO can regulate both the F-actin cytoskeleton and endothelial cell membrane stiffness. Atomic force microscopy (AFM) is a powerful tool that can be used to study plasma membrane deformability at the single cell level. As membrane stiffness is partially dependent on filamentous F-actin, the interdependence of these parameters can be studied through the combined approaches of AFM and laser scanning confocal microscopy (LSCM). In the present study, we evaluated the effects of constant or fluctuating hyperglycemia on endothelial-derived NO synthesis, the cytoskeletal contribution and endothelial cell membrane stiffness.

**Results:**

Compared to control cells cultured in low glucose (5 mM), constant (25 mM) or fluctuating (25/5 mM) high glucose significantly decreased NO release along with stiffening of endothelial cell membranes and F-actin rearrangement. The non-selective nitric oxide synthase (NOS) inhibitor, N^G^-nitro-_L_-arginine methyl ester (_L_-NAME) exerted similar effects on endothelial cells. Increasing concentrations of _L_-NAME (from 0.1 to 1 mM) exacerbated these effects in a concentration-dependent manner.

**Conclusions:**

Result from the present study suggest that stiffening endothelial cell membranes are associated with decreased NO synthesis, which was established through the F-actin cytoskeletal redistribution. The precise mechanisms of hyperglycemia-induced endothelial dysfunction require further investigation.

## Background

Vascular disease remains the major cause of increased morbidity and mortality in patients with diabetes [1]. Endothelial cell dysfunction is a significant factor contributing to the vascular complications of diabetes [2]. Hyperglycemia, particularly the fluctuation of glucose levels, causes a significant degree of oxidative stress, decreasing endothelial nitric oxide synthase (eNOS) expression [3,4], reducing nitric oxide (NO) bioavailability, and impairing NO metabolism [5].

Decreased bioavailability of endothelial-derived NO contributes to endothelial cell contraction, which is partly dependent on reorganization of the endothelial cell cytoskeleton [6-8]. Substantial evidence exists for the involvement of endogenous NO production in the regulation of the cytoskeletal F-actin structure via mobilization of intracellular Ca^2+^ by either protein kinase C (PKC) or protein kinase G (PKG) [9-11].

The actin network plays a major role in determining the mechanical properties of living cells [12]. Atomic force microscopy (AFM) can be used to study cell morphology and the micromechanical properties of both the cell surface and subsurface layers. Furthermore, recent evidence indicates that it is possible to assess the deformability of the plasma membrane at the single cell level using AFM [13]. The combined approaches of AFM and laser scanning confocal microscopy (LSCM) extends the utility of the scanning probe approach for the evaluation of cellular mechanical properties. Using immunofluorescent dyes and LSCM to dissect the three cytoskeletal elements, elasticity of the cell membrane has been found to be related to distribution of actin and intermediate filaments, with only a minor contribution from microtubules [14].

Several studies have investigated the potential relationships between NO release and endothelial cell membrane stiffness using AFM [15-17]. Recently, it was reported that acute and small changes in plasma sodium concentration can lead to a significant increase in endothelial cell membrane stiffness that associated with reduced NO release [15], while potassium ions can soften vascular endothelial cells and increase NO production [16]. However, it has not been definitively proven that endothelial-derived NO influences endothelial deformability via actin cytoskeletal reorganization. Interestingly, a number of studies have examined alterations in endothelial deformability by AFM in various physiological and pathological states [18-23]. However, the effects of stable or fluctuating hyperglycemia on endothelial cell membrane stiffness using AFM has received little attention.

In the present study we examined the effects of constant and fluctuating high glucose levels on the release of endothelial-derived NO, expression of eNOS, changes in endothelial cell membrane stiffness and the contribution of filamentous F-actin.

## Methods

### Cell culture

Human umbilical vein endothelial cells (HVUECs-12, CRL-2480) were obtained from the American Type Culture Collection, plated in 25 cm^2^ flasks (Costar, Japan) and cultured in low glucose (5 mM) Dulbecco’s Modified Eagles Medium (DMEM; GIBCO, Invitrogen, USA) supplemented with 10% fetal bovine serum (TBD, China). The flasks were incubated at 37°C in a humidified atmosphere containing 5% CO_2_. After reaching confluence, the cells were treated with 0.01% trypsin-EDTA (Amresco, USA). One ml cell suspensions containing 10^5^ cells/ml were re-seeded in 60-mm dishes (Corning, USA). The endothelial cells were allowed to attach overnight, and were exposed to the appropriate experimental conditions for seven days. In brief, eight groups of cells were prepared, each receiving the following fresh media every 24 h, respectively: 1)control group exposed to continuous low glucose (5 mM) DMEM medium; 2)continuous high glucose (25 mM) DMEM media (GIBCO, Invitrogen, USA); 3) low (5 mM) and high (25 mM) glucose media alternating every 24 h; 4) continuous low glucose (5 mM) DMEM media containing 0.1 mM non-selective NOS inhibitor N^G^-nitro-_L_-arginine methyl ester (_L_-NAME, Biyotime Co., China); 5) continuous low glucose (5 mM) DMEM media containing 0.5 mM _L_-NAME; 6) continuous low glucose (5 mM) DMEM media containing 1 mM _L_-NAME; 7) continuous osmotic control, low glucose (5 mM) DMEM media containing 20 mM mannitol (Sigma, USA); or 8) intermittent osmotic control, low glucose (5 mM) DMEM media containing mannitol (0/20 mM, alternating every 24 h).

### Nitric oxide measurement

NO was determined from the nitrite concentration in culture supernatants using the Griess reaction, as previously described [24]. Culture media was replaced every 24 h for the seven day experimental period. The harvested media was centrifuged, and the supernatant was stored at −20°C. The supernatant was pooled over seven days, and the measurement of NO was performed according to instructions for the Griess assay kits (Keygen Co., China). After addition of the Griess reagent, absorbance was measured at 550 nm using a Bio-Rad model 680 microplate reader. Nitrite concentration was determined using the formula:

$$\frac{{\mathrm{OD}}_{T}{-\mathrm{OD}}_{B}}{{\mathrm{OD}}_{S}{-\mathrm{OD}}_{B}}x100\mathrm{\mu mol}/L$$

Where OD_T _was the absorbance of the test solution; OD_B _was the absorbance of a regent blank; OD_S _was the absorbance of the 100 μmol/L standard. All experiments were performed in triplicate.

### Expression of endothelial nitric oxide synthase (eNOS)

Assays were performed using western immunoblot analyses using specific antibodies against eNOS (Genscrip, USA) and β-actin (Newmark, USA). β-actin expression was used as a loading control. One ml cell suspension was plated at a density of 10^5^/ml in 60-mm dishes (Corning, USA), allowed to attach and cultured under various conditions. Cells were subsequently washed with chilled PBS and homogenized in modified RIPA buffer (1 M Tris–HCl PH 8, 1 M NaCl, 1% Triton X-100, 0.5M EDTA PH 8, 1 M MgCl_2_, 1 mM PMSF, Boster Co., China) for 5 min. Cell lysates containing equal amounts of protein were dissolved by adding 5×SDS-PAGE sample buffer (Boster Co., China) followed by heating for 10 min. 100 μg of total protein was separated on polyacrylamide gels (the gel percentage was chosen depending on the protein being observed.) Following electrophoresis, samples were transferred to a PVDF membrane using a 25 mM Tris buffer containing 192 mM glycine and 20% methanol using a Bio-Rad mini-Blot transfer apparatus. The PVDF membranes were blocked with 5% (w/v) non-fat milk in 0.01M PBS containing 0.1% Tween 20 for 2 h at room temperature, and then incubated in the above antibodies against eNOS (1:1000 dilution) and β-actin (1: 400) overnight at 4°C. After washing with PBS/Tween (3 × 15 min), the membranes were incubated in secondary antibody (goat anti-rabbit IgG, 1:5,000 dilution, Keygen Co., China) for 2 h and washed. Protein bands were detected using chemiluminescent substrate (Pierce Biotechnology, USA) and developed on film (Kodak, USA) following a 5 min exposure. Relative band densities were quantified using densitometry with Image J software (NIH, Bethesda, MD). Data were then normalized to β-actin levels. All western immunoblot experiments were repeated at least in triplicate with separate cell preparations.

### Actin staining

Cell suspensions (total 10^5 ^cells) were plated on glass coverslips and positioned in 60 mm culture dishes. Following seven days of incubation in various media, cells were washed with chilled PBS, fixed in 3.75% formaldehyde solution, permeabilized with Triton X-100, and blocked with 1% BSA. To label F-actin, one unit of Rhodamine Phalloidin (Biotum, USA) was diluted in 200ul PBS with 1% BSA, and then added to each cover slip. The samples were stained for 20 min, and subsequently washed in triplicate with PBS. Stained samples were imaged using a Zeiss LSCM (510 META Duo Scan; Zeiss, Germany) at excitation (540 nm) and emission (565 nm) wavelengths.

### AFM imaging and measurement

Cells were planted on coverslips and cultured under various conditions for seven days. The coverslips, with seeded cells, were washed in triplicate to clear the culture media. Following preparation of the samples, an Autoprobe Cp AFM (Autoprobe CP Research, Veeco, USA) was operated in contact mode in air to obtain AFM images and perform force spectroscopy. Stiffness of the endothelial cells was determined using AFM techniques as described previously and measured with soft cantilevers, since the area of interaction between the tip and cell was larger and thus mechanically less noisy [12,15,16,25]. Silicon nitride tips (UL20B, Park Scientific Instruments) were used for all AFM measurements. The curvature radius of the tips was less than 10 nm, and the length, width and thickness of the cantilevers were 115, 30, and 3.5 μm, respectively, with an oscillation frequency of 255 kHz and a force constant of 0.01 N/m (manufacture offered). The same approach velocity was maintained to detect the endothelial cells.

Topographical morphology and deflection images of the surface of HUVECs were acquired over an area of 5-80 μm^2^. According to these images, parameters of the endothelial cells were obtained, such as Rp-v and Ra. The valley-to-peak value (Rp-v) defines the difference between the maximum and minimum values of the z coordinate on the surface of the analytical area. Ra denotes the average roughness in the analytical area. All parameters were directly generated by the software IP2.1. Subsequently, endothelial cell membrane stiffness of each chosen point was evaluated using the indentation of the local force-distance curve .

**Figure 1:**
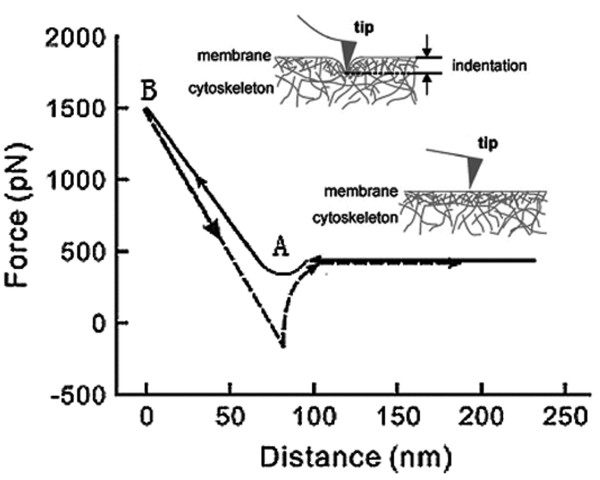
**Force-versus-distance curve.** Point A of the curve indicates the probe connects with the surface of the cells. From point A to B, the probe presses on the cell membrane and the opposing force generated along with increasing strain in the cell surface rises. At point B, the probe is ready to detach from the surface of the cell and no longer pushes against the membrane, so the opposing force reaches peak.

The AFM tip was pressed against the cell so that the membrane was indented. Cantilever deflection, measured using a laser beam when reflected from the cantilever, permitted force-distance curves of single cells (Figure 1). The slope of such curves was directly related to the force (express in Newtons), defined here as stiffness. Two different slopes could be identified depending on the depth of indentation. The initial flat slope (indentation depth to several hundred nanometers, from point A to B in Figure 1) reflects the plasma membrane stiffness, including the cortical cytoskeleton [16].

Five randomly selected endothelial cells were studied for each experimental treatment. Membrane stiffness of each cell was sampled at approximately 30 randomly chosen points on the cell center (including perinuclear) or peripheral membrane. The total average stiffness was acquired from 300 force curves.

### Statistical analyses

Results are expressed as mean ± SD. When the experimental treatments were compared to either the control group, or the group under continuous high glucose exposure, a one-way variance analyses was performed. A value of *P*<0.05 was considered statistically significant. The statistical package SPSS13.0 was used for all analyses.

## Results

### Release of endothelial-derived NO

**Figure 2:**
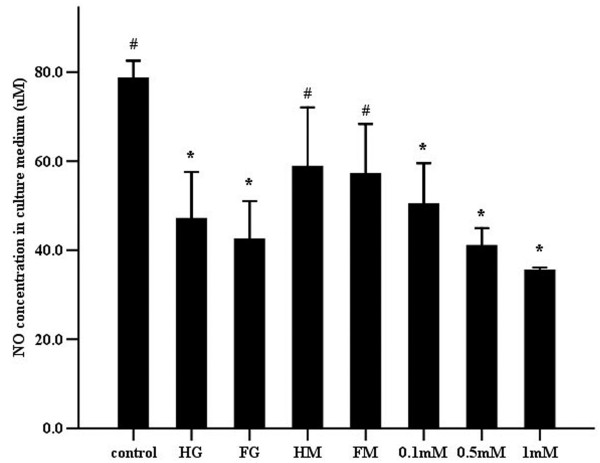
**Effects of high glucose and the NOS inhibitor **_**L**_**-NAME on the release of NO in human umbilical vein endothelial cells.** NO concentrations in the culture supernatant were measured seven days after incubation of endothelial cells in various media. Control, continuous low glucose (5mM) media; HG, continuous high-glucose (25mM) media; FG, fluctuating glucose (25/5mM, alternated every 24h) media; HM, continuous low glucose (5mM) media containing 20mM mannitol; FM, low glucose (5mM) media with 0/20mM mannitol (alternated every 24h); 0.1mM, 0.5mM and 1mM, continuous low glucose (5mM) media containing 0.1, 0.5 or 1mM _L_-NAME, respectively. Data shown in the figure are mean ± SD. “*”, *P*<0.05, compared to the control group. “#”, *P*<0.05, compared to the constant high glucose group.

Under both constant and intermittent high glucose levels, endothelial cells released significantly less NO into the culture supernatants compared to the control group. Increasing concentrations of _L_-NAME (0.1 mM to 1 mM) similarly decreased endothelial-derived NO production. Inhibition of NO release by _L_-NAME was concentration-dependent. There was no significant difference in NO production between the cells exposed to constant hyperglycemia compared to those incubated in fluctuating high glucose conditions (*P*=0.059). Constant and intermittent hypertonicity induced by 20 mM mannitol did not affect cell NO release compared to the control group (*P*=0.112) (Figure 2 and Table 1).

**Table 1:** **Effects of high glucose and NOS inhibitor **_**L**_**-NAME on the viability, NO release, eNOS expression and F-actin fluorescence intensity of human umbilical vein endothelial cells incubated in different media (as described in Figure**2**) for seven days**

**Group**	**Cell viability (Optical density)**	**NO concentration (μmol/L)**	**eNOS expression (%)**	**F-actin florescence intensity**
**Control**	**0.80±0.093**	**78.6±4.00**^**#**^	**73.7±4.40**^**#**^	**1083±261.7**^**#**^
**HG**	**0.64±0.061**	47.0±10.56^*^	50.5±0.72^*^	1538±250.7^*^
**FG**	**0.73±0.114**	42.4±8.59^*^	39.6±0.37^*#^	1566±309.3^*^
**HM**	0.50±0.106^*^	**58.7±13.34**^**#**^	57.0±1.38^*#^	**1057±316.1**^**#**^
**FM**	**0.74±0.098**	**57.1±11.24**^**#**^	56.2±1.19^*#^	**1110±385.5**^**#**^
**0.1mM**	**0.65±0.104**	50.3±9.24^*^	48.5±1.48^*^	1501±228.9^*^
**0.5mM**	**0.70±0.075**	40.9±4.00^*^	40.4±0.82^*#^	1625±344.8^*^
**1mM**	**0.59±0.156**	35.4±0.69^*^	28.3±0.29^*#^	1785±323.4^*#^

Data shown in the table are means±SD. *, *P*<0.05, compared to the control group. **#**, *P* <0.05, compared to the constant high glucose group.

### Expression of eNOS

**Figure 3:**
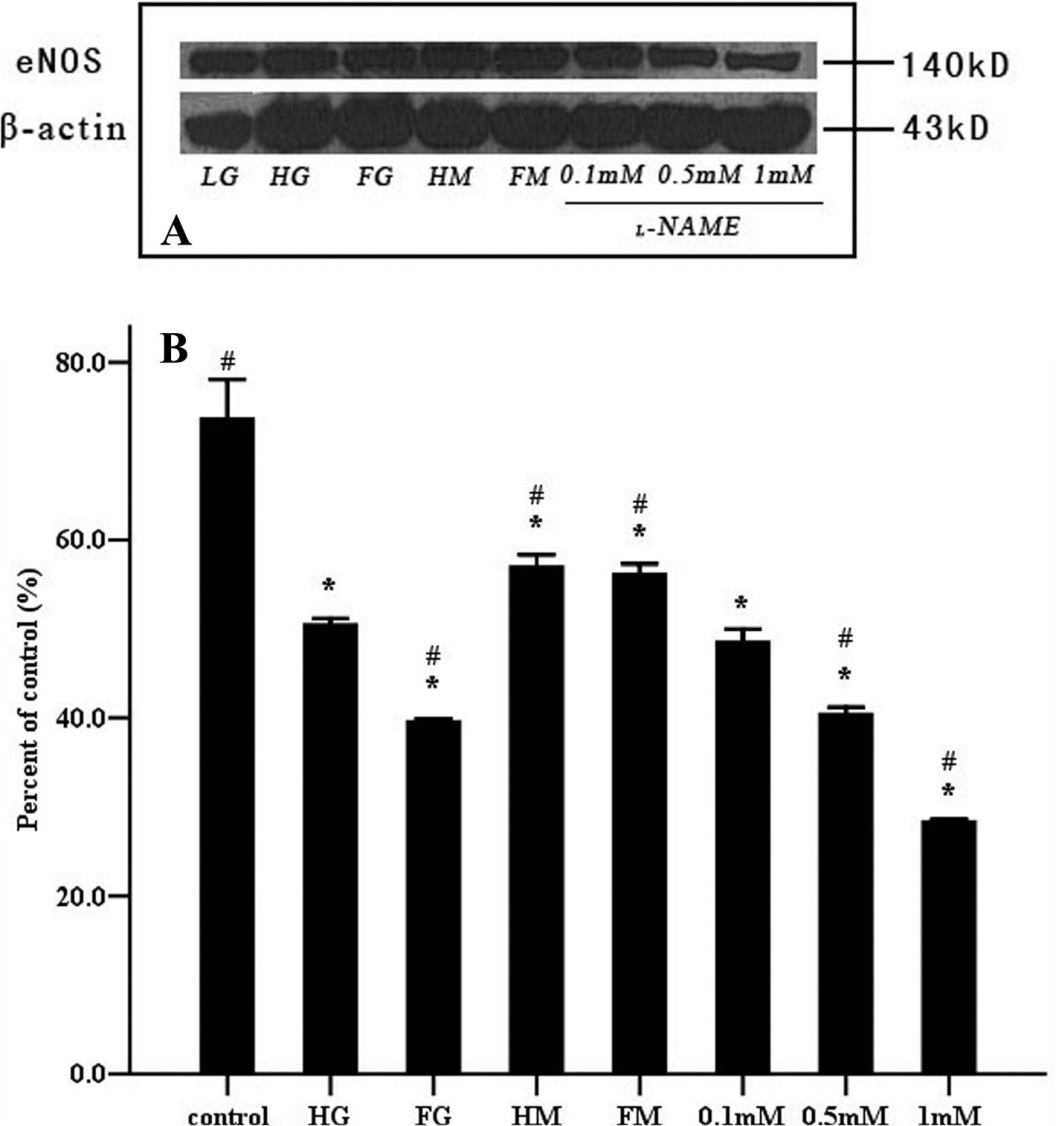
**Effects of high glucose and the NOS inhibitor **_**L**_**-NAME on the expression of eNOS in human umbilical vein endothelial cells.** The detection of the protein expression was carried out seven days after incubation of endothelial cells in various media (as described in Figure 2). (**A**) Representative western immunoblots of eNOS and β-actin. (**B**) Relative expression levels of eNOS proteins obtained using densitometric analyses of the blots, compared with those of β-actin. Data shown in the figure are mean ± SD. “*”, *P*<0.05, compared to the control group. “#”, *P*<0.05, compared to the constant high glucose group.

eNOS protein levels were determined using western immunoblot. As shown in Figure 3 and Table 1, eNOS expression was decreased significantly by constant and intermittent hyperglycemia, continuous or fluctuating hypertonicity and _L_-NAME, compared to the control group. However, the inhibitory effect of intermittent high glucose on expression appeared greater than that of constant high glucose (*P*=0.000). No significant difference in eNOS expression was observed between endothelial cells cultured under constant or fluctuating hypertonicity (*P*=0.999). Additional experiments showed that _L_-NAME (0.1 mM to 1 mM) suppressed eNOS expression in a concentration-dependent manner (Figure 3 and Table 1).

### AFM imaging and morphologic parameters

**Figure 4:**
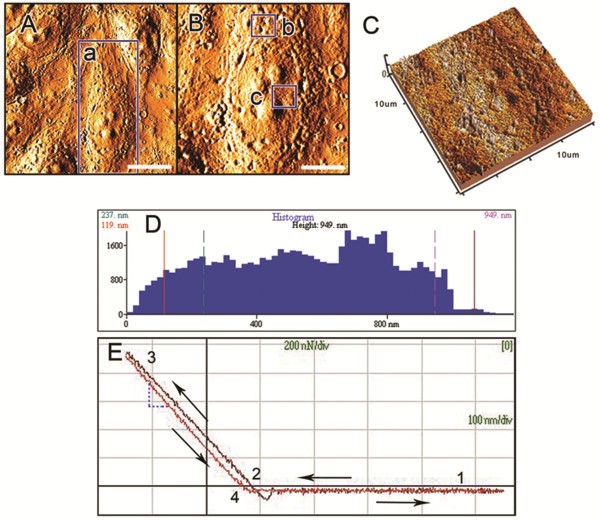
**The topological morphology (A-B) and the imaging of the third dimension (C) of endothelial cell membrane detected using AFM.** B and C were zoomed in from the region a in A. The areas b and c in B show the peripheral and central regions in the cell, where the force-distance curves were detected through the indentation force along the alteration of the distance between the tip and the studied location. Scale bar in A = 20μm; Scale bar in B-C = 10μm. The histogram (**D**) representing the morphologic characteristic was obtained from the scan of the cellular surface (**B** and **C**) with AFM. The force-distance curve (**E**) was obtained with the tip on the cell treated with continuous low glucose for seven days and presented the relative elasticity of the cell surface (approach curve, 2→3), with a noticeable adhesion force (retraction curve, 3→4).

Example figures showing topological morphology (Figure 4A and B) and height projections (Figure 4C) are shown. Scanned images were 80 μm × 80 μm (Figure 4A) and 40μm×40μm in size (Figure 4B and C), respectively. Histograms related to the morphologic characteristics were analyzed (Figures 4D), and the morphological parameters, including mean height, differences between the peak region and the valley regions (R_P-V_), maximal diameter and average roughness (Ra) were acquired and are presented in Table 2. Differences in mean height and R_P-V_ of endothelial cells exposed to low glucose containing 1 mM concentration _L_-NAME were significantly lower than control cell values. The remaining morphological data were not statistically different between cell treatments. These results indicated that the shape and volume of endothelial cells were not obviously affected under these experimental conditions allowing comparisons of the cell membrane stiffness to be performed on grossly similar cell populations.

**Table 2:** **Data of different parameters obtained by AFM in human umbilical vein endothelial cells incubated in various media (as described in Figure **2**) for seven days**

**Group**	**Mean high**	**R**_**P-V**_	**Max diameter**	**Average rough**	**Average Stiffness**
	**(nm) N=5**	**(nm) N=5**	**(nm) N=5**	**(nm) N=5**	**(pN/nm) N=300**
**control**	**629±189.0**	**1493±420.3**	**938±310.7**	**240±52.3**	**13.2±0.04**^**#**^
**HG**	**585±240.1**	**1140±450.1**	**879±386.7**	**217±83.4**	13.5±0.05*
**FG**	**479±52.1**	**923±100.9**	**900±87.5**	**199±20.7**	13.3±0.04*
**HM**	**502±91.6**	**1161±416.5**	**728±127.9**	**242±135.7**	12.6±0.80*^#^
**FM**	**483±123.4**	**1185±244.0**	**799±94.4**	**214±22.0**	**13.0±0.04**^**#**^
**0.1mM**	**358±118.6**	**777±139.4**	**707±172.6**	**129±40.8**	**13.1±0.08**^**#**^
**0.5mM**	**305±109.5**	**921±257.8**	**671±140.5**	**132±48.3**	14.0±0.07*^#^
**1mM**	211±7.6*	541±81.9*	**546±92.0**	**83±12.4**	15.0±0.08*^#^

Data shown in the table are means±SD. *, *P*<0.05, compared to the control group. **#**, *P* <0.05, compared to the constant high glucose group.

Endothelial cell membrane stiffness was estimated through the detection of the point-by-point force-distance curves (Figure 4E). Half of the 300 force curves for each group were acquired by measuring the surface of the cellular periphery and the other half in the perinuclear region (Figure 4B b and c).

**Figure 5:**
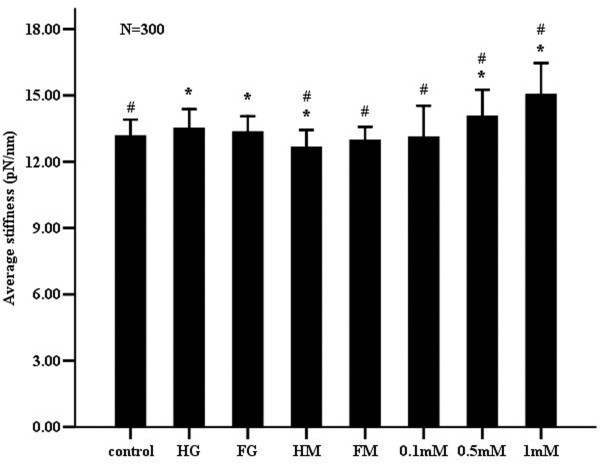
**Effects of high glucose and the NOS inhibitor **_**L**_**-NAME on the average membrane stiffness of the whole cell.** Measurement of average membrane stiffness was performed after seven days of incubating endothelial cells in various media (as described in Figure 2). The average membrane stiffness of five cells in each group was assessed through the slope of the ascending part of 300 force-distance curves detected using AFM. Data shown in the figure are mean ± SD. “*”, *P*<0.05, compared to the control group. “#”, *P*<0.05, compared to the constant high glucose group.

The average stiffness for each group was acquired from the analyses of total 300 force-distance curves (Figure 5 and Table 2). Compared with control cells, the average membrane stiffness of the cells exposed to constant and fluctuating high glucose was higher, while cell membranes treated with continuous hypertonicity were more elastic. Interestingly, the cell membrane response to increasing concentrations of _L_-NAME (0.1 mM to 1 mM) was characterized by a progressive increase in membrane stiffness. The effect of _L_-NAME was concentration-dependent. However, no differences were observed in membrane stiffness, between the cells exposed to the constant or intermittent high glucose.

### F-actin localization and quantification of the fluorescence intensity

**Figure 6:**
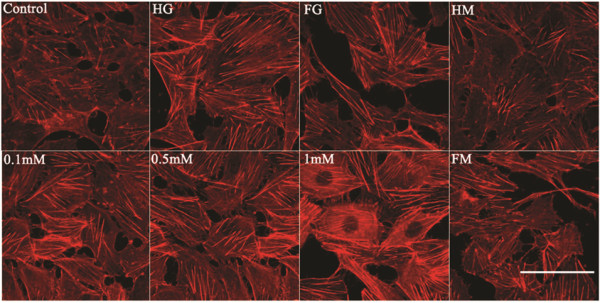
**Effects of high glucose and the NOS inhibitor **_**L**_**-NAME on F-actin cytoskeleton in human umbilical vein endothelial cells.** The detection of F-actin fluorescence was performed following seven days of incubating endothelial cells in various media (as described in Figure 2). F-actin was probed with Rhodamine Phalloidin to emit red fluorescence. Scale bar = 50μm.

**Figure 7:**
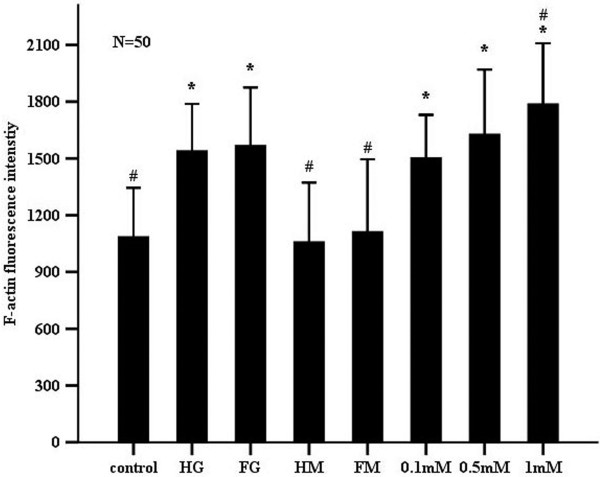
**Comparisons of the relative fluorescence intensity of F- actin in human umbilical vein endothelial cells incubated in various media (as described in Figure **2**) for seven days.** Data shown in the figure are mean ± SD. “N=50”, the average F-actin fluorescence intensity from 50 fluorescent-staining endothelial cells each group, “*”, *P*<0.05, compared to the control group. “#”, *P*<0.05, compared to the constant high glucose group.

The F-actin cytoskeleton is a major contributor to the maintenance of cellular integrity and function. F-actin in endothelial cells incubated in the varioust media for seven days was fluorescently stained and examined using LSCM (Figure 6). F-actin was clearly evident in all treatment groups with intense and filamentous staining crossing cells from one side to the other. While the structure of F-actin was unaffected by the various treatments, analyses of the fluorescence intensity indicated that expression of F-actin was altered. Exposure of cells to stable and fluctuating high glucose significantly increased the fluorescence intensity of F-actin, particularly that in the cytoplasm (Figures 6 and 7 and Table 1). Results also indicated that the increased F-actin staining induced by _L_-NAME was concentration-dependent. However, F-actin was not significantly different between constant and intermittent high glucose groups (*P*=1.000>0.05).

## Discussion

Cellular function is largely determined by its structure. Thus, cellular deformability, a mechanical property of the cellular structural organization, varies in a number of physiological processes (including cell differentiation, growth and adhesion) and in pathological states (including oxidative stress, viral infection and, parasitic infection) [13]. Recent studies have shown that alterations in endothelial cell membrane stiffness are accompanied with changes in NO production [15-17]. For example, a small physiological increase in extracellular sodium directly increases the stiffness of vascular endothelium and decreases NO release [15]. An acute increase in potassium, within a physiological range, swells and softens endothelial cells and increases the release of NO [16]. Nebivolol, a β1-receptor blocker, decreases membrane stiffness of endothelial cells, which is dependent on the increased content of NO outcome and is abrogated by _L_-NAME [17].

The above studies, however, have only focused on how experimental conditions altered endothelial cell membrane stiffness while simultaneously affecting NO synthesis. Thus, attention has not been paid to the exact effects of NO release and eNOS expression on endothelial cell membrane stiffness. Therefore, to examine the effect of NO on cell membrane stiffness, _L_-NAME was used to inhibit endogenous NO generation. Expression of eNOS (Figure 3), together with NO generation (as assessed by nitrite) (Figure 2), was quantified to clarify the relationship between cell membrane stiffness and NO metabolism. The data showed that _L_-NAME increased endothelial cell membrane stiffness (Figure 6), inhibited NO release and suppressed eNOS protein expression in a concentration-dependent manner suggesting a direct relationship between production of NO and the mechanical properties of the cell.

Endothelial cells subjected to a diabetic environment, both in vivo and in vitro, exhibit a diminished capacity for NOS-induced generation of NO [26]. Consistent with this we observed that both high glucose and _L_-NAME similarly impaired eNOS expression and reduced NO production. Exposure to high glucose has been reported to impair NO metabolism through production of oxidative stress [5,27,28]. Superoxide may interfere with the generation of NO by several mechanisms including a decrease in endothelial eNOS expression mediated by activator protein AP-1, a change in the electrophysiological state of endothelial cells and the availability of tetrahydrobiopterin, an essential cofactor of eNOS. Interestingly hyperglycemia contributes to a switch in eNOS expression in a time-dependent manner. It was found that eNOS protein expression was significantly up-regulated 12 h following exposure to high glucose concentrations (30 mM), reaching a peak at 48h (two fold increase over baseline levels) [28]. Pricci et al. also demonstrated that under high glucose conditions (20 mM), eNOS expression and nitrite/nitrate levels increase the first day, returning to normal levels at day three and diminishing thereafter [5]. Consequently, under high glucose conditions, a compensatory increase in eNOS expression appears at this early stage, thereafter declining gradually as long-term oxidative stress develops. A deleterious effect of intermittent high glucose is also mediated by free radical over-production [3,4,29].

In agreement with previous studies, incubation of endothelial cells in constant or intermittent high glucose for seven days decreased not only eNOS expression (Figure 3), but also NO concentration in the culture media (Figure 2). Interestingly, there was no significant difference in NO production between the cells exposed to constant hyperglycemia and those incubated in fluctuating high glucose conditions (Figure 2). However, intermittent high glucose reduced eNOS expression more significantly than a constant level of hyperglycemia (Figure 3). This apparent discrepancy may be related to the observation that endothelial cells exposed to intermittent high glucose are more seriously impaired than those exposed to continuous high glucose due to enhanced oxidative stress [3,4,29]. Other reports show that high glucose increases eNOS protein expression, but ultimately leads to decreased NO release [30].

Hyperglycemia plays an important role in the etiology of endothelial dysfunction [26,31]. Current data suggest that the deformability of endothelial cells detected by AFM will be affected in the presence of endothelial dysfunction [15-18,21-23,32,33]. Despite this, few studies have used AFM to explore the effects of constant and intermittent high glucose on endothelial cell membrane elasticity. In this study, analysis of 300 force-distance curves obtained using AFM (Figure 5), showed that increased average membrane stiffness of endothelial cells (in response to stable or intermittent high glucose) accompanied decreased NO release and eNOS expression (Figures 2 and 3). Simultaneously, no significant difference existed in membrane stiffness and NO release between continuous and intermittent high glucose groups (Figures 2 and 6). Thus, it is suggested that the increased membrane stiffness of endothelial cells may be affected by the change of NO release more than that of eNOS expression.

In addition to the deleterious effects on endothelial NO-dependent function, high glucose concomitantly increases extracellular osmolality, which may itself impact cellular membrane stiffness. Thus, maintained and intermittent hyperosmolality (as caused by mannitol) was used as an osmotic control for continuous and intermittent high glucose. In the presence of mannitol the release of NO (Figure 2) was unaffected while the expression of eNOS (Figure 3) was slightly decreased. Although abnormal levels of osmolality are likely harmful to endothelial cells, the absence of a NO releasing effect presumably explains why hypertonicity was not shown to increase cell membrane stiffness (Figure 5). In fact, consistent with previous observations, hypertonicity decreased membrane stiffness [21].

Actin is not only an essential component necessary for maintenance of cellular integrity and function (e.g., membrane polarity, tight junctions, cellular adhesions, and signal transduction), but also undergoes dynamic changes in response to physiological and pathological stresses, including shear stress, vascular pressure and harmful mediators [34]. Several studies have shown how decreases in NO synthesis and eNOS expression induce the redistribution of actin, especially F-actin [9-11,35,36]. NO-induced changes in F-actin filaments are proposed to be associated with mobilization of intracellular Ca^2+ ^mediated by the cGMP-dependent pathway, which is activated by cGMP-dependent protein kinase G (PKG) [11]. The stronger F-actin polymerization is also observed when endothelial cells are exposed to D-glucose pre-treated aortic smooth muscle cells, while D-manntinol has no effect on endothelial Ca^2+^ signaling [36]. TGF-β, a growth factor closely linked to diabetic microvascular complications can stimulate F-actin assembly via activation of NADPH oxidase, which is a mechanism implicated in hyperglycemia [35]. Overwhelming evidence demonstrates that actin redistribution can also regulate NO synthesis and eNOS activity not only through pre-translational mechanisms [34], but also through posttranslational mechanisms [37].

Consistent with the aforementioned studies, changes in F-actin relative fluorescence intensity in response to different media (Figures 6 and 7) were associated with decreased NO production (Figure 2) and decreased expression of eNOS (Figure 3). Treatment of endothelial cells with differing concentrations of _L_-NAME, stable and intermittent high glucose enhanced the apparent thickness of the longitudinal F-actin filaments as demonstrated by an increase in the relative fluorescence intensity of F-actin. Exposure to maintained and intermittent hyperosmolality did not alter NO production nor was, the fluorescence intensity of F-actin significantly different from the control group.

F-actin, as one of the major cytoskeletal components, clusters to form actin filaments, which are bundled and crosslinked by several actin-binding proteins into a network. The actin network plays a major role in determining the mechanical properties of living cells [12]. Furthermore, evidence suggests that depolymerization/polymerization of F-actin filaments results in a dramatic decline/enhancement in endothelial cell membrane stiffness. For example, studies by Cuerrier have demonstrated that laturculin A, an F-actin filament depolymerizing agent, causes a dramatic decline in endothelial cell membrane stiffness [23]. The variation in membrane elasticity in the various regions of endothelial cells (perinuclear or cytoplasmic membrane) is related to the distribution of cytoskeletal elements within these regions [31]. In this study, it was demonstrated that the increased average membrane stiffness of endothelial cells treated with stable/intermittently high glucose and _L_-NAME was consistent with the change in the corresponding F-actin cytoskeleton (Figures 5, 6 and 7). Therefore, it is speculated that constant and intermittent high glucose, as well as _L_-NAME treatment, may stiffen endothelial cell membranes by an alteration in F-actin expression and arrangement through the described dysfunction of NO synthesis.

## Conclusion

The present study demonstrates that constant and fluctuating high glucose levels, as well as the treatment with _L_-NAME, enhances average membrane stiffness of intact endothelial cells. Alterations in membrane stiffness and structure may be mediated by the increased expression and redistribution of the F-actin cytoskeleton, which are initiated by high glucose-induced or _L_-NAME-induced inhibition of eNOS expression and NO synthesis. Importantly, cell membrane stiffness, which is associated with the reorganization of F-actin, can be evaluated with AFM. Thus, AFM, a novel scanning probe for the study of cellular mechanical properties, can be applied to the evaluation of endothelial dysfunction under differing pathological conditions.

## Competing interest

The authors declare that they have no competing interests.

## Authors’ contribution

CX conceived of the study, participated in the design, carried out all experiments, performed the statistical analysis and drafted the manuscript. FL participated in its design and coordination and helped to draft the manuscript. HJ carried out the measurements of endothelial cell membrane stiffness using AFM. All authors read and approved the final manuscript.
